# Broad-Spectrum Antimicrobial and Antibiofilm Activity of a Natural Clay Mineral from British Columbia, Canada

**DOI:** 10.1128/mBio.02350-20

**Published:** 2020-10-06

**Authors:** Shekooh Behroozian, Sarah L. Svensson, Loretta Y. Li, Julian E. Davies

**Affiliations:** a Department of Microbiology and Immunology, University of British Columbia, Vancouver, British Columbia, Canada; b Department of Civil Engineering, University of British Columbia, Vancouver, British Columbia, Canada; Institut Pasteur

**Keywords:** antibacterial agent, clay mineral, antimicrobial clay, bacterial biofilm, fungal pathogen

## Abstract

Worldwide increases in antibiotic resistance and the dearth of new antibiotics have created a global crisis in the treatment of infectious diseases. These concerns highlight the pressing need for novel antimicrobial agents. Natural clay minerals have a long history of therapeutic and biomedical applications and have lately received specific attention for their potent antimicrobial properties. In particular, Kisameet clay (KC) has strong antibacterial activity against a variety of multidrug-resistant (MDR) bacterial pathogens *in vitro*. Here, we have extended the known spectrum of activity of KC by demonstrating its efficacy against two major fungal pathogens, Candida albicans and Cryptococcus neoformans. In addition, KC also exhibits potent activity against the opportunistic bacterial pathogen Mycobacterium marinum, a model organism for M. ulcerans infection. Moreover, aqueous KC leachates (KC-L) exhibited broad-spectrum antibacterial activity, eradicated Gram-negative and Gram-positive biofilms, and prevented their formation. The mechanism(s) underlying KC antibacterial activity appears to be complex. Adjusting KC-L to neutral pH rendered it inactive, indicating a contribution of pH, although low pH alone was insufficient for its antibacterial activity. Treatment of KC minerals with cation-chelating agents such as EDTA, 2,2′-bipyridyl, and deferoxamine reduced the antibacterial activity, while supplementation of KC-L with these chelating agents eliminated the inhibitory activity. Together, the data suggest a positive role for divalent and trivalent cations, including iron and aluminum, in bacterial inhibition by KC. Collectively, these studies demonstrate the range of KC bioactivity and provide a better understanding of the mechanism underlying its antibacterial effects.

## Abstract (precis)

The escalating emergence of multidrug-resistant (MDR) bacteria, together with the paucity of novel antimicrobial agents in antibiotic development, is recognized as a worldwide public health crisis. Kisameet clay (KC), found in British Columbia (BC), Canada, is a clay mineral with a long history of therapeutic applications among people of the First Nations. We previously reported the antibacterial activity of KC against a group of MDR clinical pathogens. Here, we demonstrate its activity against two major human-pathogenic fungal species, as well as against bacterial biofilms, which underlie many recalcitrant bacterial infections. In these studies, we also identified several geochemical characteristics of KC, such as metal ions and low pH, which are involved in its antibacterial activity. These findings provide a better understanding of the components of KC antibacterial activity and a basis for developing defined preparations of this clay mineral for therapeutic applications.

## INTRODUCTION

The discovery and development of antibiotics, once referred to as “miracle drugs,” have had a major impact in reducing morbidity and mortality due to bacterial infections (1). However, overprescription and misapplication of antibiotics have provided strong selective pressures for the widespread and escalating development of (multi)drug-resistant bacteria (2, 3). The bacterial species showing such resistance include the ESKAPE pathogens (Enterococcus faecium, Staphylococcus aureus, Klebsiella pneumoniae, Acinetobacter baumannii, Pseudomonas aeruginosa, and *Enterobacter* species), which are responsible for increasing numbers of drug-resistant nosocomial infections worldwide (4). In addition to those having acquired resistance that can be rapidly disseminated throughout populations, several emerging and opportunistic bacterial pathogens are intrinsically recalcitrant to treatment due to their physiology or growth characteristics, such as *Mycobacterium* species (5). The issue is not restricted to bacterial pathogens, as the global burden of invasive fungal infections resistant to the limited arsenal of antifungal agents is also increasing (6). These developments collectively compromise our curative power against infectious diseases, and their effects are worsened by the lack of development of new antimicrobial agents using conventional methods, which require extensive time and testing of newly discovered or newly synthesized compounds (2, 3), further emphasizing the dire need for alternative approaches to combat infectious diseases (7, 8).

Complementary and “alternative” medicines that provide novel types of antimicrobial agents could address this problem (9). Alternative strategies such as the use of phage therapy, antimicrobial peptides, therapeutic monoclonal antibodies, and antibacterial nanomedicines have been considered (9, 10). More recently, “ancient” agents such as clay minerals with demonstrated medicinal applications have become of interest (11). Clay minerals have been employed for reported curative and protective purposes (antiseptic or anti-inflammatory agents) since early history (11, 12). Aside from the topical application of medicinal clays, geophagy or geopharma (13), representing the deliberate ingestion of “earth” materials for medicinal purposes such as healing gastrointestinal disturbances and/or supplementation of minerals, has also been practiced (12, 14–16). As the most plentiful components of the Earth’s surface, clay minerals are naturally occurring nanomaterials of geological origin (17, 18). They are composed primarily of charged sheet silicates (phyllosilicates) that have specific physicochemical properties such as ultrafine grain (<2.0 μm [one dimension]), a repeating microcrystalline layered structure (stable stacks of tetrahedral silicates and octahedral metal [hydr]oxide sheets as the main building blocks), vast surface area with high potential for absorption and reactivity, and especially a high ion exchange capacity, which results from intercalation of ions and their retention in an exchangeable state (17, 19, 20). Due to their considerable chemical and mineralogical diversity and complexity, a variety of specific clay minerals have been widely employed in pharmaceutical and biomedical applications as active ingredients (21, 22), catalysts or catalyst supports (23, 24), excipients (25), or removed adsorbents or drug delivery media (26) or to enhance pharmaceutical formulations (27).

Despite the long history of their diverse applications, the potential of clay minerals in combatting infectious diseases has received specific interest only recently with the successful application of hydrated poultices of French green clay (CsAgO_2_) for healing advanced Buruli ulcer (BU) (14, 28), a chronic necrotizing cutaneous ulcer caused by M. ulcerans that is recalcitrant to traditional antibiotics (29–31). This stimulated the investigation of the antibacterial activities and physicochemical characteristics of clays in laboratory studies, which demonstrated broad-spectrum *in vitro* antibacterial activity of an iron-rich clay that had been applied to treat BU (32, 33). However, further investigation revealed that among so-called “healing” clays, relatively few deposits exhibit antibacterial properties (34). In an attempt to identify the physicochemical characteristics that make a clay antibacterial, diverse features have been identified or proposed, including clay nanoparticle size (32); low pH and redox buffering generated by hydrated clay, which supports the toxicity of specific exchanged metal ions (Fe^2+^, Cu^2+^, Zn^2+^, and Al^3+^) (35–38); or generation of antimicrobial compounds by resident bacterial species (39).

Kisameet clay (KC), formerly known as Canamin clay and B.C. peloid deposit, is a glacial clay mineral found on the north shore of Kisameet Bay on the central coast of British Columbia (BC), Canada (40, 41), and has been used by the local Heiltsuk First Nations people for purported healing properties for generations (42–44). In the mid-20th century, KC was sold as an aqueous suspension to be administered orally or topically, and anecdotal evidence suggested that the clay was effective for the treatment of ulcerative colitis, among other conditions, and had antibacterial activity (44). Our previous studies showed that aqueous suspensions of KC exhibit potent antibacterial activity against the clinically important ESKAPE pathogens *in vitro* (45). We also characterized the geochemical and microbiological features across the KC deposit to obtain insight into what might make the clay active (46). This revealed a surprising level of bacterial species richness and prevalence of iron bacteria such as *Gallionella* species near the surface of the deposit, provided molecular evidence for taxa that include medically or economically important bacteria such as *Actinomycetes*, and in general highlighted the complexity and diversity of the KC deposit. Despite these observational studies, there is limited experimental information concerning the chemical, biological, and physical composition of KC with respect to its antimicrobial properties, and its spectrum of activity is incompletely defined. Here, we show that KC is the first natural clay found to be active against fungal pathogens, as well as bacterial biofilms *in vitro*. We also provide support for hypotheses concerning its mode of action by defining some of the antimicrobial components of KC with a series of integrated microbiological, mineralogical, and chemical studies. We found that the antimicrobial properties of KC can be extracted in water, are pH dependent, and are affected by the availability of divalent and trivalent metal ions.

## RESULTS

### KC suspensions have broad-spectrum antimicrobial activity.

**FIG 1:**
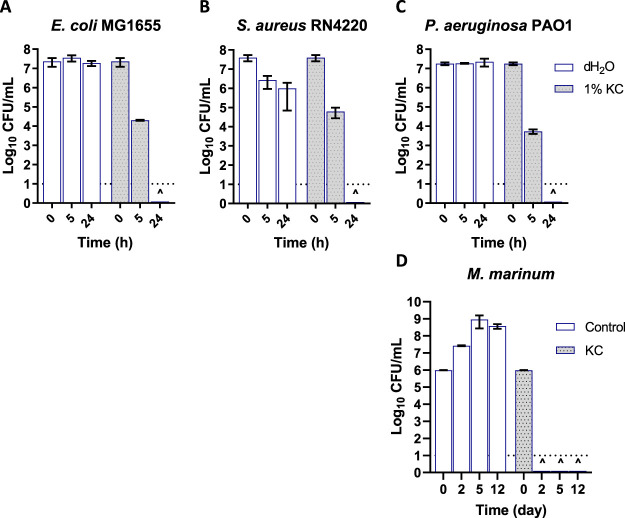
(A to C) Viability of E. coli MG1655 (A), S. aureus RN4220 (B), and P. aeruginosa PAO1 (C) after treatment with 1% (wt/vol) aqueous suspensions of KC. (D) Growth inhibition activity of 25% (wt/vol) KC against M. marinum strain M in 7H9 broth. The dotted line at log_10_ = 1 of the *y* axis represents the limit of detection for CFU. The carets (^) indicate that the levels of viable cells were below the limit of detection at the indicated time point. Error bars represent standard errors (SE) of the means of results from at least three independent replicates.

To further determine the spectrum of KC activity and provide insight into its possible mode of action, the inhibitory effect of KC against representative Gram-negative, Gram-positive, and clinically important bacteria (Escherichia coli, S. aureus, and P. aeruginosa, respectively) as well as against Mycobacterium marinum and two pathogenic fungi (Candida albicans and Cryptococcus neoformans) was investigated using *in vitro* susceptibility assays. KC exhibited antibacterial activity against all bacterial species tested (Fig. 1). Incubation of E. coli MG1655 with a 1% (wt/vol) aqueous suspension of KC (Fig. 1A) resulted in an approximately 3-log_10_ reduction in CFU after 5 h, while 24 h of treatment reduced the number of viable bacteria to below the limit of detection. In comparison, no significant decrease was observed for bacteria incubated in water alone. Similarly, S. aureus and P. aeruginosa were eliminated within 24 h (Fig. 1B and C). As was observed for E. coli, P. aeruginosa viability was reduced by ∼3.5-log_10_ in the first 5 h of exposure, while S. aureus showed a 1.5-log_10_ reduction in CFU compared to the control. KC also exhibited antibacterial activity *in vitro* against M. marinum, a model for the BU agent M. ulcerans. As shown in Fig. 1D, a 25% (wt/vol) suspension of KC in 7H9 broth eradicated all bacteria to below the limit of detection within 2 days of treatment *in vitro*. The same inhibitory effect was observed on 7H10 agar media supplemented with 25% (wt/vol) KC (data not shown).

**FIG 2:**
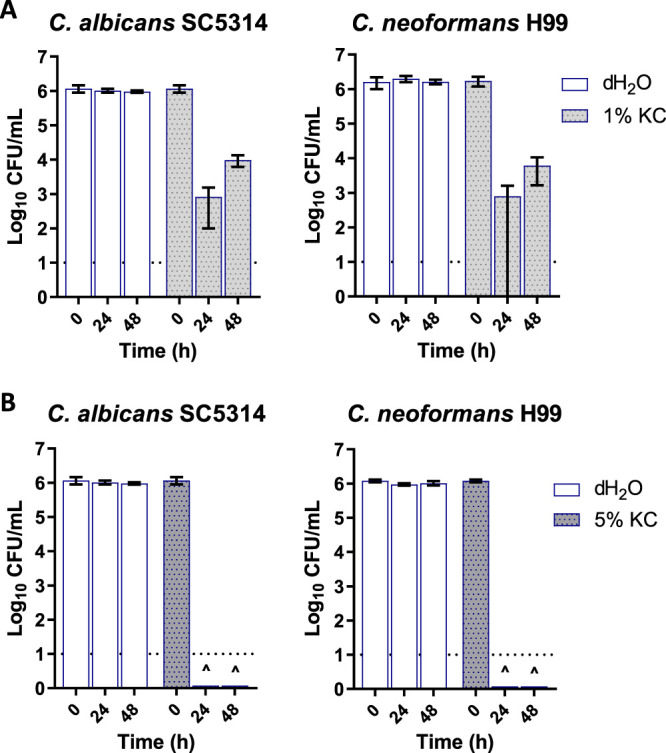
Viability of fungal strains C. albicans SC5314 (left) and C. neoformans H99 (right) after treatment with 1% (wt/vol) (A) or 5% (wt/vol) (B) aqueous suspensions of KC at 30°C. The dotted line at log_10_ = 1 represents the limit of detection. The carets (^) indicate that the levels of viable cells were below the limit of detection at the indicated time point. Error bars represent standard errors (SE) of the means of results from at least three independent replicates.

To extend the spectrum of KC to other clinically relevant microbes, we tested its activity against two major pathogenic fungi. C. albicans is the most prevalent human-pathogenic fungal species (47). It resides as a constituent of the healthy microbiota but can overgrow and cause superficial and mucosal infections and/or disseminate into the bloodstream and opportunistically cause invasive infections in internal organs (47, 48). We also investigated the activity of KC against C. neoformans, an emerging encapsulated fungal pathogen which infects both immunocompromised and immunocompetent individuals, causing diseases ranging from cutaneous lesions to systemic and possibly fatal diseases such as pulmonary and meningeal infections (49–51). KC in 1% (wt/vol) aqueous suspensions inhibited both fungal strains at 37°C (data not shown) but did not do so completely at 30°C (Fig. 2A) within 48 h. However, suspensions of 5% KC reduced the number of CFU to below the detection limit within 24 h of treatment at 30°C (Fig. 2B).

### KC antibacterial activity can be extracted with water.

**FIG 3:**
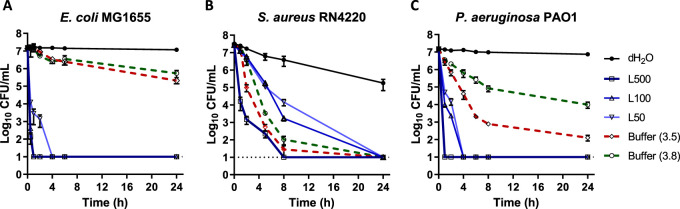
Antibacterial activity of KC aqueous leachates L50, L100, and L500 against E. coli MG1655 (A), S. aureus RN4220 (B), and P. aeruginosa PAO1 (C) compared to low-pH phosphate buffers. The dotted line at log_10_ = 1 of the *y* axis represents the limit of detection for CFU. Error bars represent standard errors (SE) of the means of results from at least three independent replicates. The key is shared by all three panels. The pH of each solution is shown in parentheses.

Exchangeable metal ions have been reported to be responsible for the activity of some antibacterial clays (35–37). We and others have previously found that the mineralogical composition and chemical properties of KC differ from those of other known natural antibacterial clay minerals (43, 46, 52). Next, we investigated if the exchangeable/soluble fraction of the clay might be involved in KC activity. To that end, aqueous leachates (L50, L100, and L500, made from 50, 100, and 500 mg/ml KC suspensions in water, respectively), from which clay solids are excluded by centrifugation and filtration, were prepared. Assays against E. coli MG1655, S. aureus RN4220, and P. aeruginosa PAO1 revealed strong antibacterial activity (with complete eradication after 1 to 4 h for two Gram-negative strains and 8 to 24 h for S. aureus), which increased with the amount of clay used to make the leachates (Fig. 3). L500 and L100 eliminated E. coli after 1 h, as did L50 within 4 h (Fig. 3A). This shows that a component responsible for the antibacterial activity of KC is water soluble.

### Low pH is required for antibacterial activity of KC aqueous leachates.

As KC leachates (KC-L) are acidic and acidity increases with the amount of clay used in their preparation (pH 3.5 to 3.8; see Table S1 in the supplemental material), we examined the effect of the low pH on viability by comparing the three KC leachates to comparable phosphate buffers (pH 3.5 and 3.8). The low-pH buffers caused a less than 1-log_10_ decrease in the viability of E. coli in 4 h, suggesting that leachate activity was not solely due to acidic pH. P. aeruginosa exhibited higher sensitivity to low-pH buffers than E. coli, as its viability was reduced to 1-log_10_ to 2.5-log_10_ within 4 h (Fig. 3A and C). However, similarly to the results seen with E. coli, in contrast to the acidic buffers, L500 showed complete killing of P. aeruginosa in 1 h whereas L100 and L50 exhibited full bactericidal activity within 4 h. The viability of S. aureus treated with L500 was ∼0.8-log_10_ to ∼1.8-log_10_ less than that seen with a corresponding pH control within the first 5 h (Fig. 3B). However, as S. aureus is intrinsically more sensitive to pH than E. coli or P. aeruginosa, the difference between the leachate and the pH control was significant (*P* < 0.05) for only the first 2 h after treatment. No viable bacteria were recovered after 24 h in either leachate or buffer. We conclude that while low pH is a characteristic of KC leachates, it was not solely responsible for their activity under the conditions tested.

10.1128/mBio.02350-20.3 TABLE S1 Chemical analysis of Kisameet clay by inductively coupled plasma optical atomic emission spectroscopy (ICP-OES). Download Table S1, DOCX file, 0.03 MB. Copyright © 2020 Behroozian et al. 2020 Behroozian et al. This content is distributed under the terms of the Creative Commons Attribution 4.0 International license.

**FIG 4:**
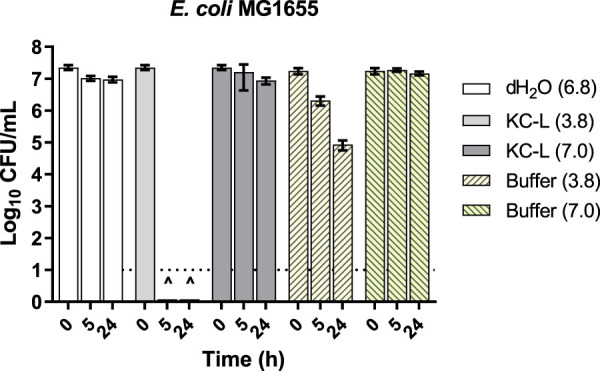
Effect of pH on antibacterial activity of KC aqueous leachates. From L50 (pH 3.8), a sample of leachate with pH altered to 7.0 was prepared. Levels of viability of E. coli MG1655 treated with these leachates or with 100 mM phosphate buffer at equal pH were compared. The dotted line at log_10_ = 1 of the *y* axis represents the limit of detection for CFU. Error bars represent standard errors (SE) of the means of results from at least three independent replicates. The pH of each solution is shown in parentheses.

Certain exchangeable metal ions are more soluble at low pH, and we hypothesized that the acidic nature of KC leachates (KC-L) may be required for their activity. We therefore determined if adjusting the pH of “active” or “inactive” KC-L could change its activity. Comparative viability assays against E. coli MG1655 treated with native and pH-altered leachates showed that increasing the pH of the leachate from 3.8 to 7.0 rendered it ineffective (Fig. 4). Native samples of KC from different depths of the deposit had different pHs (lower pH at the top of the deposit) (46). We next tested if the activity of leachates prepared from vertical core samples with higher pH could be enhanced (see Fig. S1 in the supplemental material). Confirming a role for low pH in activity, we found that lowering the pH of KC aqueous leachates prepared from vertical core samples with a higher native pH (6.1 to 7.1) to 4.4 enhanced their antibacterial activity. Importantly, pH 4.4 buffer again did not affect viability to the same levels as those seen with pH-adjusted core leachates.

10.1128/mBio.02350-20.2 FIG S1 Antibacterial activity of aqueous leachates, prepared from 5% (wt/vol) aqueous suspensions of vertical core samples collected from depths of 0 to 28 feet from site Kis3 in Kisameet Bay deposit (46), against E. coli MG1655. Download FIG S1, PDF file, 0.1 MB. Copyright © 2020 Behroozian et al. 2020 Behroozian et al. This content is distributed under the terms of the Creative Commons Attribution 4.0 International license.

### Divalent and trivalent metal cations are required for KC antibacterial activity.

**FIG 5:**
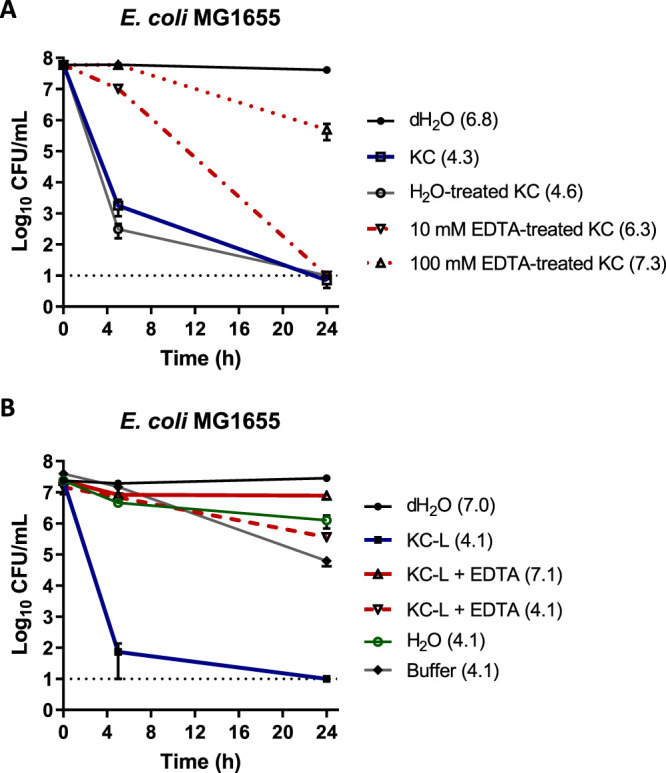
Viability of E. coli MG1655 in 1% (wt/vol) aqueous suspension of KC treated with EDTA (A) and of KC aqueous leachate (KC-L) with EDTA and pH adjustment (B). The dotted line at log_10_ = 1 of the *y* axis represents the limit of detection for CFU. Error bars represent standard errors (SE) of the means of results from at least three independent replicates. The pH of each solution is shown in parentheses.

The observed pH dependence of leachate activity suggests that acid-soluble metal ions might also contribute to KC antibacterial activity. Elemental analysis of KC aqueous leachates by inductively coupled plasma optical emission spectrometry (ICP-OES) revealed that the five most abundant metallic elements in the leachates were Al, Ca, Fe, Mg, and Na (Table S1; see also Text S1 in the supplemental material). The role of metal cations in KC mineral and leachate activity was tested using various cation chelators. EDTA, a broad-spectrum chelator, sequesters a variety of metal ions, including Al^3+^, Ca^2+^, Cu^2+^, Fe^2+^, Mg^2+^, and Ni^2+^ (53). As shown in Fig. 5A, KC prewashed with 10 mM EDTA exhibited (∼4.5-log_10_) reduced inhibitory activity compared to KC washed with H_2_O alone within 5 h of incubation. However, both EDTA-treated KC and H_2_O-treated KC reduced the bacterial viability to below the detection limit within 24 h of treatment. In contrast, prewashing of dry KC with a higher-concentration (100 mM) EDTA solution completely eliminated its antibacterial activity against E. coli. While this finding suggests that removal of metal ions might reduce KC activity, it is also possible that an increase in pH caused by EDTA treatment also influenced the activity of the treated clay. Therefore, we directly treated aqueous leachates (whose pH could be more easily controlled than suspensions) with EDTA. Our studies showed that antibacterial activity of the leachates was also eliminated by the addition of 10 mM EDTA (final concentration) (Fig. 5B). Since the addition of EDTA to L50 increased its pH from 4.1 to 7.1 (Table 1), we adjusted the pH of L50 treated with EDTA to the initial pH of the leachate (4.1). This did not restore activity, suggesting that exchangeable divalent and/or trivalent cations are important for KC toxicity.

**TABLE 1:** pH of KC aqueous suspensions and leachates treated with EDTA, BPY, or DFO^*a*^

KC suspension	pH	KC aqueous leachate	pH
KC 1% aqueous suspension	4.3	KC-L (L50)	4.1
H_2_O-treated KC	4.6	KC-L + 10 mM EDTA	7.1
10 mM EDTA-treated KC	6.3	KC-L + 10 mM EDTA—adjusted pH	4.1
100 mM EDTA-treated KC	7.3	KC-L + 1 mM BPY	5.0
10 mM BPY-treated KC	4.8	KC-L + 1 mM BPY—adjusted pH	4.1
10 mM DFO-treated KC	3.3	KC-L + 1 mM DFO	3.4
10 mM DFO-treated KC—adjusted pH	4.2	KC-L + 1 mM DFO—adjusted pH	4.1

a BPY, 2,2′-bipyridyl; DFO, deferoxamine.

10.1128/mBio.02350-20.1 TEXT S1 Supplemental methods: elemental analysis by inductively coupled plasma optical atomic emission spectroscopy (ICP-OES). Download Text S1, DOCX file, 0.02 MB. Copyright © 2020 Behroozian et al. 2020 Behroozian et al. This content is distributed under the terms of the Creative Commons Attribution 4.0 International license.

**FIG 6:**
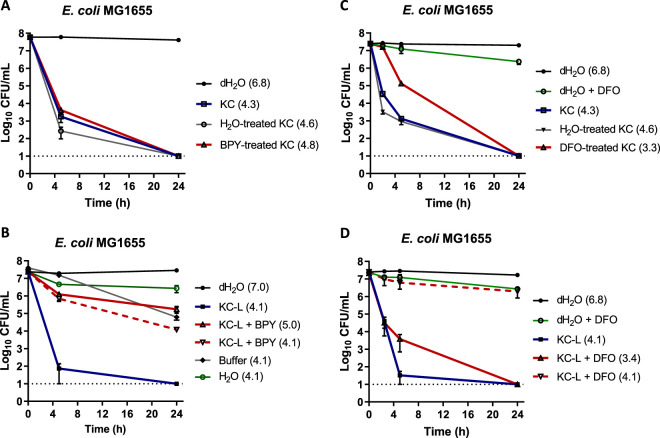
Viability of E. coli MG1655 in 1% (wt/vol) aqueous suspension of KC prewashed with 2,2′-bipyridyl (BPY) (A) or 10 mM deferoxamine (DFO) (C) and of KC leachate (KC-L) treated with BPY and pH adjustment (B) or 1 mM DFO (D). The dotted line at log_10_ = 1 of the *y* axis represents the limit of detection for CFU. Error bars represent standard errors (SE) of the means of results from at least three independent replicates. The pH of each solution is shown in parentheses.

Significant amounts of Fe were detected in leachates (Table S1), and other studies also reported previously that KC is an iron-rich clay (42, 46). Therefore, the effect of two iron-specific chelators, 2,2′-bipyridyl (BPY) and deferoxamine (mesylate) (DFO), on KC activity was investigated. BPY is a bidentate chelator that predominantly binds Fe^2+^, while DFO as a hexadentate chelating agent preferentially chelates Fe^3+^ (54–57). Dry KC prewashed with 10 mM BPY was initially less active (∼1.5-log_10_ increase in the viability of E. coli compared to H_2_O-treated KC) within the first 5 h of incubation. However, after 24 h, the level of activity was identical to that of nontreated KC (Fig. 6A). When 1 mM BPY (final concentration) was directly added to aqueous leachate (L50) instead, the antibacterial activity decreased to 4-log_10_ compared to the original leachate, and adjusting the pH to the initial value (4.1) did not restore the activity (Fig. 6B). In contrast to BPY, DFO is a strong Fe^3+^ chelator (stability constant of ∼31.0) (Table S2). Panel C of Fig. 6 shows that prewashing of dry KC with 10 mM DFO reduced its antibacterial activity against E. coli about 2-log_10_ compared to H_2_O-treated clay within 5 h of incubation. When L50 was directly supplemented with 1 mM DFO, activity was almost completely eliminated (Fig. 6D). Together, these results support the idea of a role for ferric iron in the activity of KC. However, as DFO is able to form strong complexes with other trivalent ions, notably Al^3+^ and Cr^3+^ (58, 59), and to chelate some divalent cations such as Co^2+^, Cu^2+^, Fe^2+^, Ni^2+^, and Zn^2+^ with lower stability constants (Table S2) (60, 61), the results do not exclude possible roles of these cations in the bactericidal activity of KC.

10.1128/mBio.02350-20.4 TABLE S2 Metal stability constant (pK_s_) of chelators. Download Table S2, DOCX file, 0.02 MB. Copyright © 2020 Behroozian et al. 2020 Behroozian et al. This content is distributed under the terms of the Creative Commons Attribution 4.0 International license.

### KC leachates prevent biofilm formation and eradicate preformed biofilms *in vitro*.

**FIG 7:**
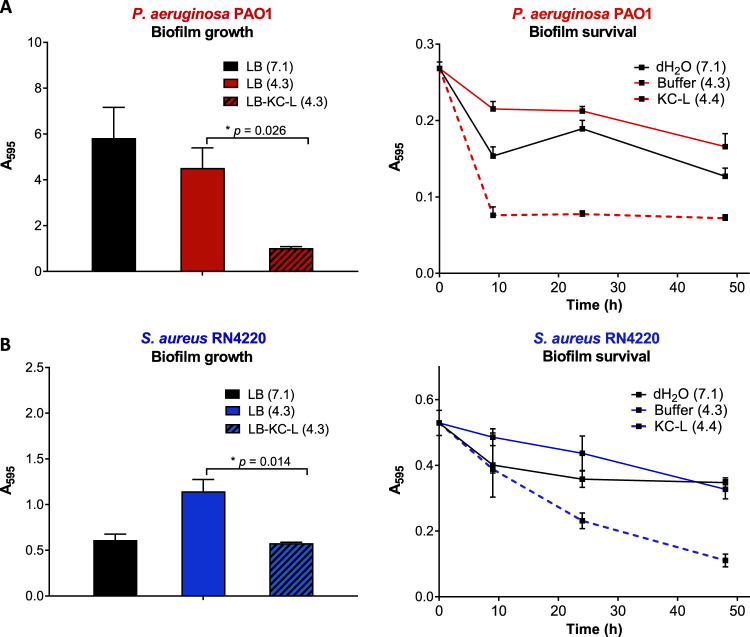
Effect of KC leachates on biofilm formation and growth (left) and survival of preformed biofilm (right) in crystal violet assays of P. aeruginosa PAO1 (A) and S. aureus RN4220 (B). Error bars represent standard errors (SE) of the means of results from at least three independent replicates. The pH of each solution is shown in parentheses.

As KC aqueous leachates were found to be antibacterial, this provided us with the opportunity to test KC activity in biofilm microplate assays without interference from clay particles. The ability of KC leachate L500 to interfere with biofilm formation or eradicate preformed biofilms was thus investigated using P. aeruginosa and S. aureus. For growth experiments (Fig. 7, left panels), a leachate prepared in lysogeny broth (LB) was compared to a control of LB broth adjusted to a similar low pH (4.3). Two days after inoculation, staining and quantification of P. aeruginosa biofilms showed that the low-pH broth had a modest effect (approximately 25% reduction) on biofilm formation (Fig. 7A, left panel). In contrast, approximately 4-fold less was observed with cultures grown in the LB-leachate (*P* < 0.05) than with the pH control. For S. aureus (Fig. 7B, left panel), biofilm formation increased when the pH of the medium was decreased from 7.0 to 4.3. Compared to the pH control, significantly (*P* < 0.05) reduced biofilm mass was seen in the cultures grown in the presence of KC leachate.

To assess the ability of KC to eradicate or disassemble bacterial consortia, preformed biofilms of P. aeruginosa and S. aureus were treated with KC leachates. The amount of biofilm remaining (as shown by crystal violet staining) was determined at different time points after initiation of treatment. For P. aeruginosa (Fig. 7A, right panel), we observed a small reduction of the amount of stained biofilm material recovered in deionized water (dH_2_O, pH 7.1). A stronger effect on the amount of stained biofilm material measured was observed with pH 4.3 buffer than with water alone. In contrast, treatment of the P. aeruginosa biofilms with KC leachate reduced the amount of stained biofilms to 30% of pretreatment levels in 10 h. KC leachates also reduced the amount of stained biofilm material in 10-h S. aureus biofilms to approximately 30% of the amount measured at time zero (Fig. 7B, right panel), although the effect was slower (requiring approximately 24 h to reach 30% of the original level seen with stained biofilm material) than for P. aeruginosa. After 48 h, the levels of S. aureus biofilms were further reduced to approximately 25% of the initial material. Together, these observations confirm that KC acts against preformed biofilms and inhibits their growth.

## DISCUSSION

Antibiotics have revolutionized modern medicine and saved countless lives; however, the rapid emergence of resistant pathogens worldwide warns of a return to the preantibiotic era (1, 8). As bacterial evolution toward resistance has overwhelmed traditional antibiotic repertoires, “alternative” sources have received increasing attention (8). Although natural products have in fact been the source of or inspiration for many of the antibacterial and therapeutic agents used currently in the clinic (62), they nonetheless require rigorous scientific analyses to confirm their efficacy and to define their complex nature and active principal components. Here, we have described studies of a naturally occurring clay mineral, Kisameet clay, which has potent, broad-spectrum antimicrobial properties *in vitro*. The antimicrobial assays presented in this study confirm the potent inhibitory activity of KC against a variety of bacterial strains, including M. marinum as a model for M. ulcerans, as well as against two fungal pathogens, C. albicans and C. neoformans, which are pathogenic fungi mostly causing infections in immunocompromised patients (47, 49). Modern medicine has paradoxically increased the number of immunocompromised patients who are more vulnerable to fungal infections characterized as “hidden killers” (63, 64). We found that these two fungal pathogens exhibited increased susceptibility to KC at 37°C compared to 30°C. The temperature-related sensitivity to antimicrobial therapy has been reported previously (65, 66).

Among the different bacterial strains tested, Gram-negative bacteria were more sensitive to both KC aqueous suspensions and leachates than Gram-positive bacteria, which reflects what was observed previously with the ESKAPE pathogens (45). KC is also strongly active against multidrug-resistant (MDR) and extensively drug-resistant opportunistic pathogens, such as members of the Burkholderia cepacia complex, isolated from cystic fibrosis patients (S. Behroozian, J. E. A. Zlosnik, and J. Davies, unpublished data). S. aureus in turn exhibited lower susceptibility to KC leachates, with a prolonged time (8 to 24 h, compared to 1 to 4 h for Gram-negative strains) required for complete elimination of cells and also low tolerance to low-pH buffers (Fig. 3B). The significant halotolerance of S. aureus, with its well-documented ability to grow at salt concentrations greater than 1 M, together with its more rigid cell wall structure, may explain, in part, this difference (67, 68). The broad-spectrum antibacterial activity of KC leachates suggests that the soluble/exchangeable fraction of KC is involved in the activity such as has been reported for other clay minerals (35, 36, 38, 69). Cunningham et al. previously reported the antibacterial activity of aqueous leachates of two antibacterial clay mixtures and demonstrated that the *in vitro* antibacterial activity of the clay might depend on chemical desorption of specific metal ions from the surface of the clay particles (35).

In addition, KC exhibited bactericidal activity against biofilms. Many bacteria exist in the environment as “multicellular” biofilm communities, which exhibit enhanced resistance to antimicrobials (70). Biofilm formation is a survival strategy enabling microorganisms to adapt to hostile environments and to tolerate antibiotics and the activity of the immune system (71, 72). Prevention of biofilm formation is therefore an important anti-infective strategy, and antimicrobials must be able to penetrate biofilms and be active against their residents. Approximately 65% of all human infections are caused by biofilm-forming bacteria (70). Recently, Kalan et al. reported that chronic nonhealing wounds are hosts to polymicrobial communities that form biofilms and interfere with treatment and healing (73). Despite the well-documented increased tolerance of biofilm bacteria to antibiotics (10 to 100 times), it has been reported that biofilm formation cannot provide resistance against metal cation toxicity (70, 72, 74).

Studies with other clays suggested that cations may underlie the antibacterial activity of mineral clays (35–38). The enhanced survival of E. coli in KC-L supplemented with metal chelators suggests that cations are major contributors to the antibacterial activity of KC leachates. The reported hierarchy of EDTA relative binding affinities for metal ions is as follows: Cr^2+^ > Fe^3+^ > Cu^2+^ > Pb^2+^ > Zn^2+^ > Cd^2+^ > Co^2+^ > Al^3+^ > Fe^2+^ > Mn^2+^ > Ca^2+^ > Mg^2+^ (60). While both DFO and BPY have been reported to form stable complexes with Co^2+^, Cu^2+^, Fe^2+^, Ni^2+^, and Zn^2+^ with different pK_s_ (metal stability constant) values, only DFO is able to chelate Fe^3+^, Al^3+^, and Cr^3+^ significantly (53, 55, 57, 59). DFO forms 1:1 complexes with Fe^2+^ or Fe^3+^, whereas three molecules of BPY are necessary to coordinate with one Fe ion (54, 75). This might explain the finding that the potency of EDTA and DFO in eliminating the activity of treated KC leachates is higher than that of BPY. These observations, together with the pH studies, provide insight into the mechanism of leachate action and are consistent with the hypothesis of the role of acid-soluble metal ions in the antibacterial activity of KC.

Cation exchange studies have revealed that the antibacterial activity of French green clay (CsAgO_2_) can be repressed, suggesting a role for exchangeable cations in bactericidal activity (32). Otto and Haydel (2013) demonstrated that mineral clay samples exhibiting cidal activity contained higher concentrations of chemically accessible metal ions (Fe^2+^, Cu^2+^, and Zn^2+^) than those with less activity (36). Different metal ions cause damage to microbial cells as a consequence of membrane function impairment, interference with nutrient assimilation, production of oxidative stress by producing reactive oxygen species and depleting antioxidants, protein dysfunction, and enzyme inactivation (76). While we likewise have provided evidence, using cation-specific chelating agents, that similar metal ions might be responsible for the activity of KC, additional analyses are required to identify the specific mechanisms underlying this effect and to determine whether other characteristics of KC act in concert, or even synergistically, with metal ions. For example, analysis of the transcriptional response of sensitive bacteria upon KC treatment or screens for resistant/tolerant mutant strains might provide evidence for the active component(s) of KC, as well as for their molecular targets. Moreover, the effects of KC against diverse clinically relevant species, such as the ESKAPE pathogens, *Burkholderia* spp., Gram-positive pathogens, mycobacteria, and fungi, should also be tested to determine if the molecular mechanisms underlying the activity of this complex material are the same for different microbial physiologies.

The potent antibacterial action of KC against human pathogens of clinical importance (45), together with its antifungal and anti-biofilm activities, suggests that KC has potential as an antimicrobial agent against antibiotic-resistant microbes. Identification of the molecular mechanisms underlying the activity of KC (including both soluble fraction and mineral particles) should allow the development of more-defined, homogeneous, consistent, and stable preparations of KC for further therapeutic applications. As clay minerals are heterogeneous mixtures, defining the specific characteristics of KC needed for antibacterial activity is essential to standardize and improve chemical consistency for medicinal application. This might also provide insight into what makes other mineral clays antibacterial and might ultimately guide the development of modified mineral-based antimicrobial derivatives for therapeutic applications with increased activity or decreased toxicity. Our results provide a first source for defining and controlling the antibacterial activity of KC and may lead to clay modifications and derivatives for clinical applications. Together, these observations expand the applications of KC as an antimicrobial agent, set the stage for creating defined preparations for different therapeutic applications, and so provide insights for the identification of other mineral clays with antimicrobial activity.

## MATERIALS AND METHODS

### Clay samples.

The unprocessed clay used in this study was supplied in its original hydrated form as collected from the field by Kisameet Glacial Clay Inc. The sample was previously shown to have potent antibacterial activity (45). Clay was dried in a vacuum desiccator at room temperature or by heating in an oven at 60°C. Dry KC samples were ground by a mortar and pestle and autoclaved at 121°C for 1 h before testing. Measurement of pH was performed on equilibrated suspensions of 1 g clay in 10 ml deionized water (dH_2_O) or aqueous leachates using a VWR-SB20 pH meter.

### Microbial strains and growth conditions.

All bacterial strains (E. coli K-12 MG1655, S. aureus RN4220, and P. aeruginosa PAO1) (77–79) were obtained from the laboratory collection. E. coli and P. aeruginosa were grown in LB broth or on LB agar. S. aureus RN4220 was grown in tryptic soy broth (TSB) or on tryptic soy agar (TSA). All bacterial liquid cultures were incubated at 37°C with gentle orbital rotary shaking. M. marinum strain M (80), kindly provided by Patricia Champion, University of Notre Dame, was grown in 7H9 broth or on 7H10 agar (Difco) supplemented with 1% OADC (oleic acid, albumin, dextrose, and catalase) (Middlebrook) and 0.2% glycerol. C. albicans SC5314 (ATCC MYA-2876) was also from the laboratory collection, while C. neoformans H99 was kindly supplied by James Kronstad, University of British Columbia. C. albicans was grown in TSB or on TSA plates and C. neoformans in YPD (1% [wt/vol] yeast extract, 2% peptone, 2% dextrose) broth or on YPD agar at 30°C.

### KC suspension and aqueous leachate preparations.

Aqueous suspensions of KC with 1% or 5% (wt/vol) concentrations were prepared by suspending 10 or 50 mg of dry, ground, autoclaved clay in 1 ml sterile dH_2_O. KC aqueous leachates (L50, L100, and L500) were obtained by adding 1, 2, or 10 g of autoclaved KC to 20 ml of sterile dH_2_O, resulting in 5%, 10%, or 50% (wt/vol) aqueous suspensions, respectively. After continuous agitation for 24 h at room temperature, suspensions were centrifuged at 25,000 rpm for 2 h at 4°C to separate insoluble minerals from the leachate solution. The supernatants were clarified and sterilized by passage through a 0.22-μm-pore-size filter (Millipore).

### Antimicrobial assays of KC aqueous suspensions and leachates.

The antimicrobial activity of KC was determined as described previously (45). Briefly, overnight cultures of bacterial or yeast strains were diluted into fresh growth medium to a concentration of ∼10^7^ CFU/ml, based on the optical density at 600 nm (OD_600_), and were incubated at 37°C (bacteria and fungi) or 30°C (fungi) with gentle rotary mixing at 200 rpm to reach the mid-exponential phase of growth based on growth curves (data not shown). Cells were harvested by centrifugation, rinsed once in sterile phosphate-buffered saline (PBS) (pH 7.4), and resuspended either in a suspension of KC in dH_2_O (1% [wt/vol] and 5% [wt/vol] for bacterial and fungal strains, respectively) or in dH_2_O only (to study the viability of organisms in the absence of KC) at an initial cell concentration of ∼10^7^ CFU/ml. Suspensions were incubated at the appropriate temperature as described above with shaking at 200 rpm to prevent sedimentation and to provide contact with the clay minerals or aqueous leachates. M. marinum strain M was used as a model for M. ulcerans. Mid-exponential-phase growth of bacteria in 7H9 broth supplemented with 1% OADC and 0.2% glycerol was followed by treatment with 250 mg/ml KC and incubation at 30°C. The inhibitory activity was studied during 12 days by determining the viability of bacteria treated with KC compared to the control without treatment.

Antibacterial assays with different leachates were performed similarly. For leachate experiments, phosphate buffers with a low pH comparable to that of KC leachates were used as negative controls. Viability was determined by 10-fold serial dilution plating of aliquots removed at the start of experiments and at two time points following exposure to clay suspensions (5 and 24 h for bacterial cells and 24 and 48 h for fungal strains) and six different time points within 24 h of exposure for aqueous leachate experiments.

### pH adjustment of aqueous leachates.

A KC aqueous leachate, L50, was prepared as described above. The initial pH was measured, and samples of the leachate were adjusted to a pH of 7.0 using 1 M NaOH. The leachate was then sterilized by filtration through a 0.22-μm-pore-size filter before use. The antibacterial activity of the pH-adjusted leachate was compared to the activity seen with the initial leachate and 100 mM phosphate buffers by testing in a viability assay with E. coli as described above.

### Preparation of cation-depleted KC suspensions.

KC mineral (1 g dry clay per 20 ml, 5% [wt/vol]) was treated with cation chelator solutions (10 mM or 100 mM aqueous EDTA [Sigma] or 10 mM aqueous 2,2′-bipyridyl [BPY; Sigma] [final concentration]) or with dH_2_O with continuous shaking for 24 h at room temperature. To treat KC with deferoxamine (mesylate) (DFO, Sigma), a KC mineral suspension (5% [wt/vol]) was washed twice with DFO (10 mM final concentration) or dH_2_O with continuous shaking for 3 h at room temperature. KC particles were then washed twice with dH_2_O to remove remaining chelators and collected by centrifugation (25,000 rpm for 2 h), dried, ground using a mortar and pestle, and autoclaved for 1 h at 121°C.

### Treatment of KC leachate with chelating agents.

A KC aqueous leachate (L50) (pH 4.1) was prepared as described above and treated by addition of EDTA (10 mM final concentration), BPY (1 mM final), or DFO (1 mM final). EDTA and BPY solutions were less acidic than L50, at pH 7.1 and at pH 5.0, respectively, so aliquots of each were restored with 1 M HCl to the original pH and then sterilized by filtration through a 0.22-μm-pore-size membrane. DFO (1 mM final) was more acidic than L50 at pH 3.4, so an aliquot of L50 plus DFO was adjusted with 1 M NaOH to restore the original pH and then filter sterilized.

### Measurement of biofilm formation and eradication *in vitro*.

To determine the effect of KC on growth of P. aeruginosa PAO1 and S. aureus RN4220 biofilms, a leachate in LB broth (50% [wt/vol], final pH 4.3) was prepared as described above for aqueous leachates, except that LB was used instead of dH_2_O. A control of LB broth adjusted to pH 4.3 with 2-(*N*-morpholino)ethanesulfonic acid (MES) buffer (50 mM) (LB-MES) was also prepared. Strains were cultured aerobically overnight in LB and diluted to an OD_600_ of 0.002 into fresh LB (pH 7.0), LB-MES (pH 4.3), or a leachate of KC prepared from 5% (wt/vol) KC–LB (pH 4.3). Two-hundred-microliter volumes of these bacterial suspensions were dispensed into the wells of 96-well polystyrene microtiter plates and were then incubated statically at 37°C under aerobic conditions. After 2 days, wells were stained with 250 μl of 1% (wt/vol) crystal violet (Sigma)–100% ethanol for 10 min. Wells were rinsed three times with water, and adhered crystal violet-stained material (biofilm) was dissolved in 250 μl destaining solution (40% [vol/vol] ethanol, 5% [vol/vol] acetic acid) and quantified by reading absorbance at 595 nm (A_595_). For analysis of viability of preformed biofilms after treatment with KC leachates, biofilms in LB were grown for 2 days. The medium was then removed and replaced with deionized water (pH 7.1) or phosphate buffer (pH 4.3) or KC leachate (L500, pH 4.35). Plates were then incubated aerobically at 37°C and stained with crystal violet as described above at 0, 10, and 24 h posttreatment. Experiments were performed with 3 to 6 wells per treatment per time point.
