# Supplementary material for: Broad-Spectrum Antimicrobial and Antibiofilm Activity of a Natural Clay Mineral from British Columbia, Canada
Source: mBio. 2020 Oct 6;11(5):e02350-20. doi: 10.1128/mBio.02350-20 (PMC7542368; doi:10.1128/mBio.02350-20)
Supplement: TABLE S1 [file mBio.02350-20-st001.docx]

**TABLE S1** Chemical analysis of Kisameet clay by inductively coupled plasma optical atomic emission spectroscopy (ICP-OES).

| **Element** | **L50** | | **L100** | | **L500** | | **MDL** | **Analyte** |
| --- | --- | --- | --- | --- | --- | --- | --- | --- |
|  | **µg/L** | **µM** | **µg/L** | **µM** | **µg/L** | **µM** | **µg/L** | (emission nm) |
| **Ag** | BDL | *-* | BDL | *-* | BDL | *-* | 2.4 | Ag 328.068 |
| **Al** | 5,538.64 | *205.27* | 13,888.38 | *395.05* | 28,211.20 | *1,045.56* | 8.3 | Al 396.153 |
| **As** | 14.60 | *1.16* | 3.0 | *0.76* | 1.4 | *0.71* | 36.1 | As 188.979 |
| **B** | BDL | *-* | BDL | *-* | BDL | *-* | 1 |  |
| **Ba** | 6.69 | *0.05* | 6.61 | *0.09* | 5.90 | *0.04* | 0.6 | Ba 233.527 |
| **Be** | 5.42 | *0.60* | 9.72 | *1.21* | 16.34 | *1.81* | 0.5 | Be 313.107 |
| **Ca** | 77,680.57 | *1,938.24* | 114,861.44 | *2,865.95* | 117,550.81 | *2,933.05* | 10 | Ca 317.933 |
| **Cd** | BDL | *-* | BDL | *-* | BDL | *-* | 1.9 | Cd 228.802 |
| **Co** | 92.16 | *1.56* | 129.66 | *2.20* | 215.06 | *3.65* | 2.6 | Co 228.616 |
| **Cr** | 4.5 | *0.09* | 2.1 | *0.04* | 1.2 | *0.02* | 1.1 | Cr 267.716 |
| **Cu** | 103.68 | *1.63* | 154.30 | *2.86* | 208.90 | *2.95* | 4.2 | Cu 327.393 |
| **Fe** | 24,219.78 | *433.70* | 28,681.31 | *505.53* | 5,0754.72 | *908.85* | 7.7 | Fe 238.204 |
| **K** | 2,808.73 | *71.84* | 3153.69 | *80.66* | 4024.71 | *102.94* | 50.0 | K 766.490 |
| **Li** | 87.88 | *12.66* | 180.33 | *25.98* | 299.24 | *43.12* | 0.3 | Li 670.784 |
| **Mg** | 43,577.07 | *1,792.93* | 62,636.82 | *2,577.12* | 74,293.20 | *3,056.71* | 652.1 | Mg 285.213 |
| **Mn** | 2,792.86 | *50.84* | 4,188.46 | *76.24* | 6,615.16 | *120.41* | 13.6 | Mn 257.610 |
| **Mo** | BDL | *-* | BDL | *-* | BDL | *-* | 5.8 | Mo 202.031 |
| **Na** | 9,284.50 | *403.85* | 12,870.88 | *559.85* | 20,962.53 | *911.81* | 50.0 | Na 589.592 |
| **Ni** | 51.37 | *0.88* | 87.25 | *1.49* | 113.48 | *1.93* | 4.0 | Ni 231.604 |
| **P** | BDL | *-* | BDL | *-* | BDL | *-* | 50.0 | P 213.617 |
| **Pb** | BDL | *-* | BDL | *-* | BDL | *-* | 2.9 | Pb 220.353 |
| **S** | 35,910.2 | *1119.92* | 101,410.2 | *3,162.65* | 301,609.2 | *9,406.18* | 50.0 | S 181.975 |
| **Sb** | BDL | *-* | BDL | *-* | BDL | *-* | 20.4 | Sb 206.836 |
| **Se** | BDL | *-* | BDL | *-* | BDL | *-* | 37.5 | Se 196.026 |
| **Si** | 5,623.54 | *200.23* | 8,380.38 | *298.39* | 9,339.26 | *332.54* | 10 | Si 251.611 |
| **Sn** | BDL |  | 10.8 | *0.09* | 282.0 | *2.38* | 10.6 | Sn |
| **Sr** | 271.50 | *3.099* | 396.3082 | *4.52* | 453.7433 | *5.18* | 4.2 | Sr 407.771 |
| **Ti** | BDL | *-* | BDL | *-* | BDL | *-* | 0.8 | Ti 334.940 |
| **Tl** | BDL | *-* | *BDL* | *-* | BDL | *-* | 36.7 | Tl 190.801 |
| **V** | BDL | *-* | BDL | *-* | BDL | *-* | 65.4 | V 290.880 |
| **Zn** | 953.30 | *14.58* | 459.82 | *7.03* | 571.97 | *8.75* | 1.1 | Zn 206.200 |
| **pH** | ***3.77-3.82*** | | ***3.60-3.64*** | | ***3.47-3.51*** | |  |  |

Aqueous leachates of KC, L50, L100, and L500, were prepared from different concentrations of aqueous suspensions of KC (50, 100, and 500 mg/mL, respectively). Double deionized H_2_O used for making leachate was used as a blank.

B, Cd, Mo, P, Pb, Sb, Se, Ti, Tl, and V were all below the detection limits for three samples of leachates.

MDL: Minimal detection limit; BDL: Below detection limit
