# Supplementary figures and images for: Broad-Spectrum Antimicrobial and Antibiofilm Activity of a Natural Clay Mineral from British Columbia, Canada
Source: mBio. 2020 Oct 6;11(5):e02350-20. doi: 10.1128/mBio.02350-20 (PMC7542368; doi:10.1128/mBio.02350-20)

FIG S1

Behroozian et al.

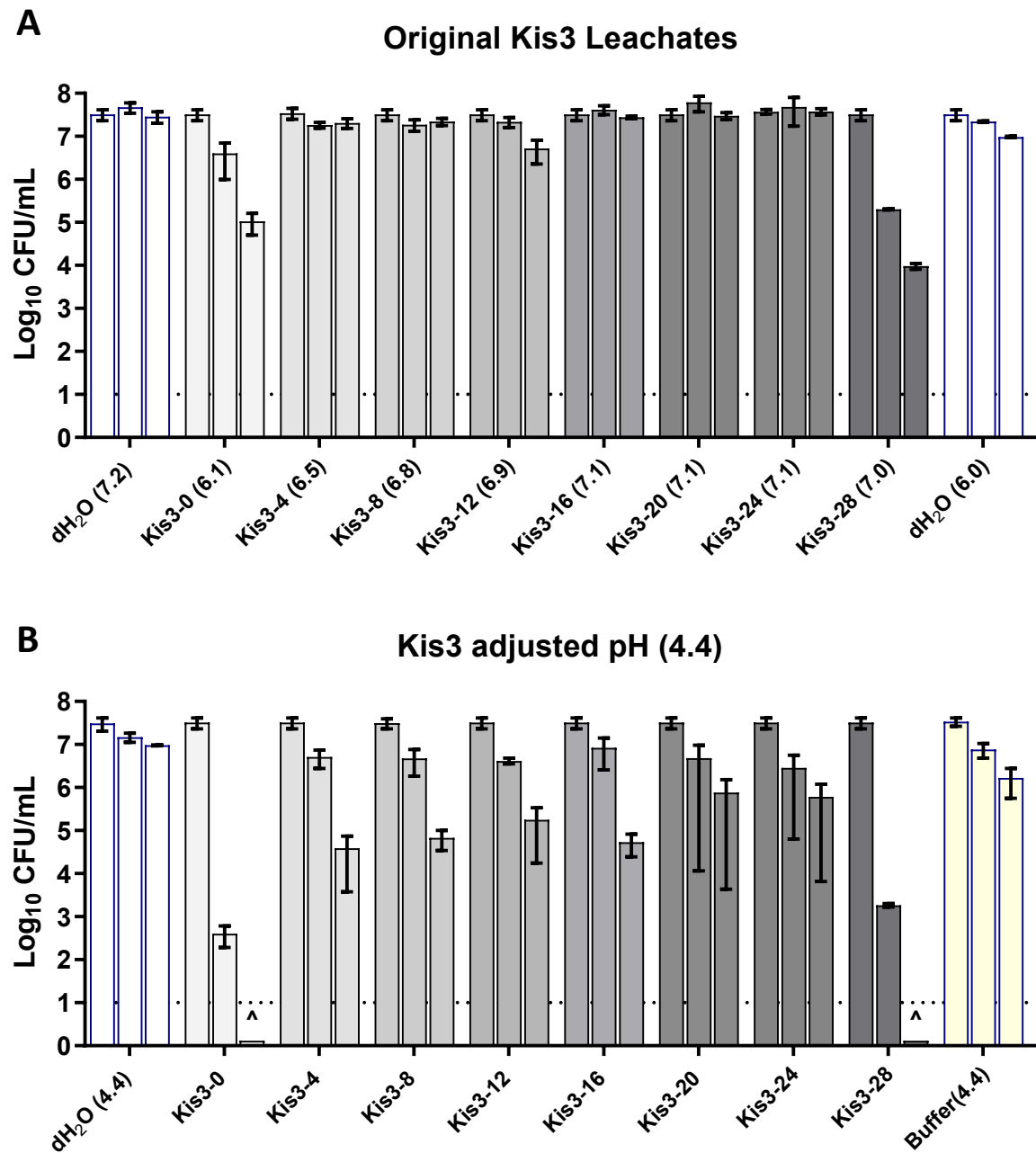

Supplement: FIG S1 [file mBio.02350-20-sf001.pdf]
