# Supplementary material for: Broad-Spectrum Antimicrobial and Antibiofilm Activity of a Natural Clay Mineral from British Columbia, Canada
Source: mBio. 2020 Oct 6;11(5):e02350-20. doi: 10.1128/mBio.02350-20 (PMC7542368; doi:10.1128/mBio.02350-20)
Supplement: TEXT S1 [file mBio.02350-20-s0001.docx]

**SUPPLEMENTAL MATERIAL**

**SUPPLEMENTAL METHOD S1 (TEXT S1)**

**Elemental analysis by inductively coupled plasma optical atomic emission spectroscopy (ICP-OES)**. The elemental compositions of KC leachates (L50, L100, and L500) were analyzed by ICP-OES using a Perkin Elmer Optima 7300 DV spectrometer equipped with a Scott spray chamber and Gem Tip Cross-Flow nebulizer. Calibration standards were prepared from certified multi-element stock solutions (Multi Element Calibration Standard 3, Perkin Elmer Inc., 4400-010 Quality Control Standard-21 elements, Atomic Spectroscopy Standard) except for Si, S, and P which were single element standards. A working calibration curve of at least seven measurements was prepared by diluting stock solutions in 0.5% nitric acid. Three KC leachate samples, blank (dH_2_O used for preparing leachates), and standards were also diluted with 0.5% nitric acid solution until their response was determined to be within the calibration range. Internal standards (Y) were added to both standards and samples prior to analysis.

**FIG S1 LEGEND:**

**FIG S1** Antibacterial activity of aqueous leachates, prepared from 5% (wt/vol) aqueous suspensions of vertical core samples collected from site Kis3 in Kisameet Bay deposit (Ref 46), from depth 0-28 ft against *E. coli* MG1655.

A) Antibacterial activity of original aqueous leachates with initial pH 6.1-7.1 compared to pH controls after 0, 5, and 24 h of incubation.

B) Increased antibacterial activity of Kis3 leachates after lowering the pHs to 4.4 compared to pH controls.

The dotted line at log_10_ =1 of the *Y* axis represents the limit of detection for CFU. ^ indicates that viable cells were below the limit of detection at that time point. The pH of each solution is shown in parentheses.

Complete cidal activity in 24 h of treatment was observed for adjusted leachates of samples collected from 0 ft and 28 ft. The antibacterial activity of other samples improved leading to a 1-4-log_10_ more reduction in the viability of bacteria.

These results show that lowering the pH of core sample aqueous leachates to 4.4 increased the antibacterial activities leading to complete eradication of bacteria for two samples (Kis3-0, Kis3-28). In addition, a 1-4 log_10_ decrease in the viability of bacteria was observed for other leachates with adjusted pH.
