# Supplementary material for: Broad-Spectrum Antimicrobial and Antibiofilm Activity of a Natural Clay Mineral from British Columbia, Canada
Source: mBio. 2020 Oct 6;11(5):e02350-20. doi: 10.1128/mBio.02350-20 (PMC7542368; doi:10.1128/mBio.02350-20)
Supplement: TABLE S2 [file mBio.02350-20-st002.docx]

**TABLE S2** Metal stability constant (pK_s_) of chelators

| **Metal ion** | **EDTA** | **DFO** | **BPY** |
| --- | --- | --- | --- |
| **Al^3+^** | 16.1 | 22.0 | - |
| **Ca^2+^** | 10.6-11.0 | 2.0 | - |
| **Cd^2+^** | 16.4 | - | - |
| **Co^2+^** | 16.1 | 11.0 | 16.1 |
| **Cr^3+^** | - | 21.0 | - |
| **Cu^2+^** | 18.4 | 14.0 | 17.5 |
| **Fe^2+^** | 14.4 | 10.0 | 17.5 |
| **Fe^3+^** | 25.1 | 30.6-31.0 | - |
| **Mg^2+^** | 8.7-9.0 | 4.0 | - |
| **Mn^2+^** | 13.4 | - | - |
| **Ni^2+^** | 18.4 | 10.0 | 20.1 |
| **Pb^2+^** | 18.3 | - | - |
| **Zn^2+^** | 16.1-16.6 | 11.0-11.1 | 13.7 |

(Refs 53, 55, 57, and 59)
